# Solution conformations of Zika NS2B-NS3pro and its inhibition by natural products from edible plants

**DOI:** 10.1371/journal.pone.0180632

**Published:** 2017-07-10

**Authors:** Amrita Roy, Liangzhong Lim, Shagun Srivastava, Yimei Lu, Jianxing Song

**Affiliations:** Department of Biological Sciences, Faculty of Science, National University of Singapore, Singapore, Singapore; Russian Academy of Medical Sciences, RUSSIAN FEDERATION

## Abstract

The recent Zika viral (ZIKV) epidemic has been associated with severe neurological pathologies such as neonatal microcephaly and Guillain-Barre syndrome but unfortunately no vaccine or medication is effectively available yet. Zika NS2B-NS3pro is essential for the proteolysis of the viral polyprotein and thereby viral replication. Thus NS2B-NS3pro represents an attractive target for anti-Zika drug discovery/design. Here, we have characterized the solution conformations and catalytic parameters of both linked and unlinked Zika NS2B-NS3pro complexes and found that the unlinked complex manifested well-dispersed NMR spectra. Subsequently with selective isotope-labeling using NMR spectroscopy, we demonstrated that C-terminal residues (R73-K100) of NS2B is highly disordered without any stable tertiary and secondary structures in the Zika NS2B-NS3pro complex in the free state. Upon binding to the well-characterized serine protease inhibitor, bovine pancreatic trypsin inhibitor (BPTI), only the extreme C-terminal residues (L86-K100) remain disordered. Additionally, we have identified five flavonoids and one natural phenol rich in edible plants including fruits and vegetables, which inhibit Zika NS2B-NS3pro in a non-competitive mode, with *K*i ranging from 770 nM for Myricetin to 34.02 μM for Apigenin. Molecular docking showed that they all bind to a pocket on the back of the active site and their structure-activity relationship was elucidated. Our study provides valuable insights into the solution conformation of Zika NS2B-NS3pro and further deciphers its susceptibility towards allosteric inhibition by natural products. As these natural product inhibitors fundamentally differ from the currently-known active site inhibitors in terms of both inhibitory mode and chemical scaffold, our finding might open a new avenue for development of better allosteric inhibitors to fight ZIKV infection.

## Introduction

Zika virus (ZIKV) was a neglected, mosquito-borne flavivirus because of its assumed small geographical spread and mild clinical symptoms [1] such as fever, headache, rashes and etc [2]. The first biological ZIKV sample was isolated from a sentinel rhesus monkey in the Zika Forest of Uganda in 1947 [3], and it was later found ZIKV is transmitted to humans by Aedes mosquitoes. However since 2007, large epidemics of Asian genotype ZIKV have been reported around the world [4,5]. Recently it is estimated that one-third of the world population might be at risk of infection [6]. The rapid rise in ZIKA infection is compounded by the ease of vertical [7] and sexual human-to-human transmissions [8]. Recent studies have associated ZIKV infection with other diseases: Guillain-Barré syndrome and microcephaly in newborn infants of mothers infected with ZIKV during pregnancy [7,9–11], thrombocytopenia [12], multiple-organ failures [13], and possibly male infertility [14]. Consequently WHO has declared a public health emergency for ZIKV infection [15]. ZIKV represents a significant challenge to the public health of the whole world but unfortunately there is no available effective vaccine or therapy so far.

ZIKV has a single-stranded positive sense RNA genome of 10.7 kb, belongs to the flavivirus genus which also contains viruses causing dengue, yellow fever, West Nile, Japanese encephalitis and tick-borne encephalitis [6,16]. ZIKV shares a high degree of sequence and structural homology with other flaviviruses particularly dengue virus, thus resulting in immunological cross-reactivity [17]. Current Zika outbreaks are largely localized within dengue-endemic areas, it is thus proposed that preexisting dengue-induced antibodies may enhance Zika infection by antibody-dependent enhancement (ADE), a factor that makes the vaccine approaches extremely challenging [17]. While several recent studies focused on the possibility of developing neutralizing antibodies against ZIKV [18–20], it may take quite a while before such neutralizing antibodies for ZIKV enter clinical trials.

ZIKV genome is translated into a single ~3,500-residue polyprotein, which is further cleaved into 3 structural proteins and 7 non-structural proteins [16]. The correct processing of the polyprotein is essential for replication of all flaviviruses, which requires both host proteases and a viral NS2B-NS3 protease (NS2B-NS3pro) and this property makes the flaviviral NS2B-NS3pro a well-established target for developing antiviral drugs [16,21–32]. While there are studies testing the inhibition effects of certain drugs on NS2B-NS3pro such as bromocriptine [33], boronate [34], or small compounds inhibitors [35], we are interested in new chemical scaffolds that are found in the edible foods. Our focus here is on whether natural edible products contain small molecules that have some inhibitory effects on the enzymatic activities of ZIKV NS2B-NS3pro. Thus in the present study, we have characterized the solution conformations and catalysis of two enzymatically active Zika NS2B-NS3pro: one with NS2B and NS3pro linked by extensively used (Gly)_4_-Ser-(Gly)_4_ sequence; and another with NS2B and NS3pro unlinked. Subsequently, we identified several small molecular inhibitors from edible plants: five flavonoids and one natural phenol which were shown to inhibit Zika NS2B-NS3pro in a non-competitive mode. Further molecular docking suggests that these molecules inhibit Zika NS2B-NS3pro by binding to a pocket on the back of the active site and allosterically affect the structure-activity property of Zika NS2B-NS3pro. As such, identification of these natural product inhibitors here night provide new avenues for development of allosteric inhibitors against ZIKV infection.

## Results

### Cloning, expression and purification of linked and unlinked Zika NS2B-NS3pro

Based on the sequence alignment with NS2B and NS3pro of the Dengue serotype 2 we previously characterized [21], the corresponding Zika sequences were identified for the NS2B (S1A Fig) and NS3pro (S1B Fig) of the Asian Zika strain 8375 (GenBank ID: KU501217.1). From synthetic genes with *E*. *coli* preferred codons, we amplified and subsequently cloned the DNA fragments into His-tagged expression vectors, which encode the isolated NS2B (48–100) with the transmembrane regions deleted (S1A Fig); as well as isolated NS3 (14–185) (S1B Fig). We also constructed a Zika protease with NS2B and NS3pro linked by a (Gly)_4_-Ser-(Gly)_4_ sequence which was extensively used for functional and structural characterization of flaviviral NS2B-NS3pro complexes [23–27].

The linked NS2B-NS3pro protein was detected in the pellet of *E*. *coli* cells with induction of 1 mM isopropyl β-D-thiogalactopyranoside (IPTG) for four hours at 37°C, while a portion of recombinant protein was found to be in supernatant with induction of 0.2 mM IPTG overnight at 18°C. Consequently, we purified the linked NS2B-NS3pro by Ni^2+^-affinity column under two conditions: the soluble form directly from the supernatant under native condition, but the insoluble form from inclusion body under denaturing condition, which was easily refolded by overnight dialysis against PBS buffer (pH 7.4) with 10 mM β-mercaptoethanol (column 2 of S1C Fig). The linked complexes without His-tag were successfully obtained by cleavage with thrombin covalently linked to beads, followed by FPLC purifications on a gel filtration column (HiLoad 16/60 Superdex 200) (column 3 of S1C Fig). Nevertheless, the linked Zika NS2B-NS3pro complexes purified directly from supernatant and from the refolding were indistinguishable as judged from both enzymatic activity and biophysical characterizations by CD, fluorescence and NMR.

On the other hand, the wild-type NS2B and NS3pro domain are not covalently linked. Furthermore, it has been previously demonstrated that only the unlinked Dengue NS2B-NS3pro manifested well-dispersed NMR spectra [30,31]. Therefore, we went on further to express the isolated Zika NS2B and NS3pro. While the NS3pro protein was found to be in inclusion body, the NS2B was detected in supernatant. So we purified them by Ni^2+^-affinity column under denaturing condition for NS3pro and under native condition for NS2B. We first attempted to refold NS3pro alone without NS2B by dialyzing NS3pro overnight against PBS buffer (pH 7.4) with 10 mM β-mercaptoethanol, but all NS3pro protein precipitated during dialysis and no enzymatic activity could be detected, suggesting that Zika NS3pro domain also needs NS2B to fold correctly, as previously observed on all other flaviviral NS2B-NS3pro [21–31]. However, using the same protocol, the mixture of NS2B and NS3pro was easily refolded into the soluble complex (column 2 of S1D Fig), was subjected to further cleavage of His-tag and the final FPLC purification (column 3 of S1D Fig). As small peptides diffuse significantly and thus usually cannot be seen in the SDS-PAGE system we used here, we checked the presence of the NS2B peptide in the finally purified unlinked NS2B-NS3pro complex by the reverse-phase (RP) high pressure liquid chromatography (HPLC) with an analytic C8 column. The HPLC profile clearly showed that two peaks exist: one with the shorter retention time is for NS2B while another with the longer retention time is for NS3pro (S1F Fig).

### Biophysical characterization

**Fig 1:**
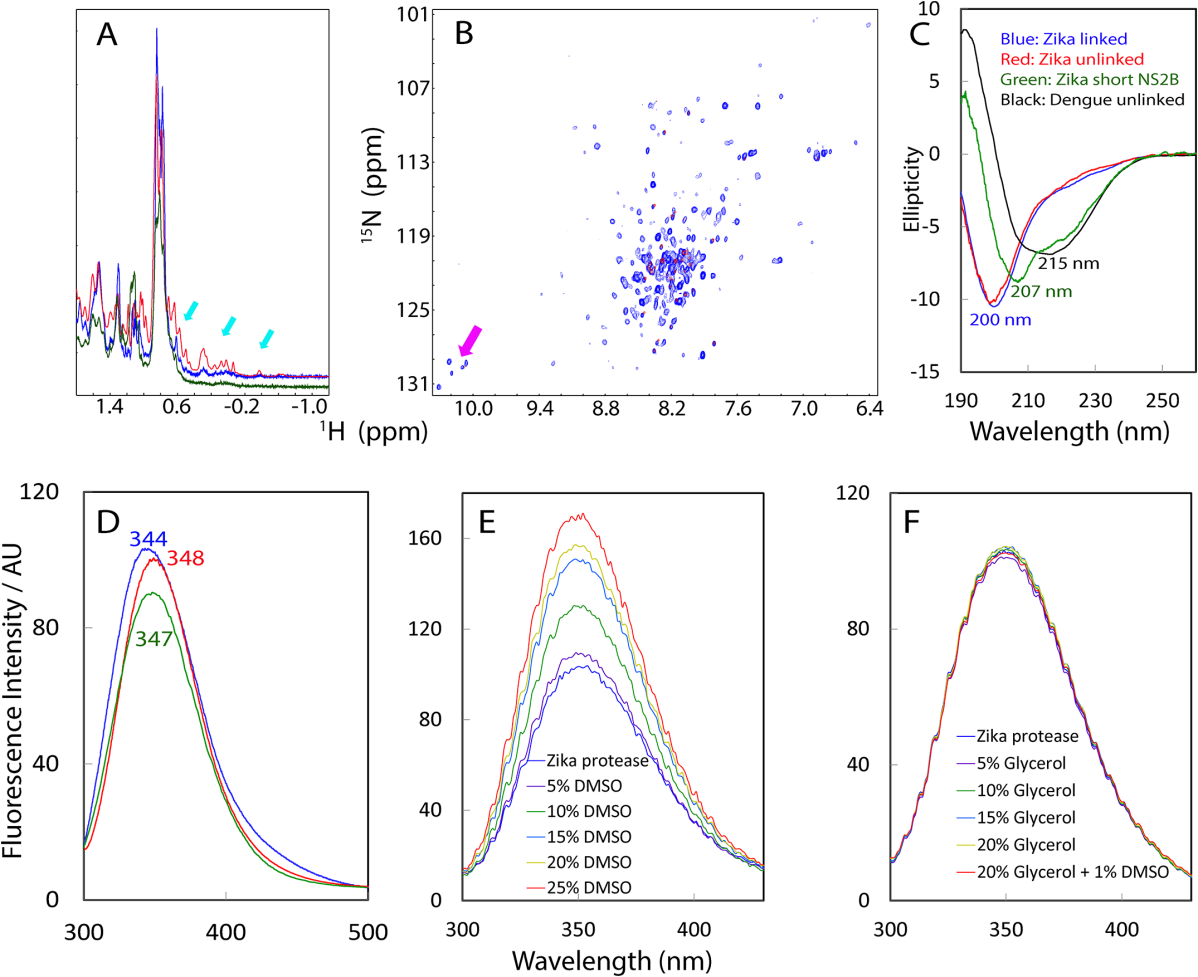
Biophysical characterization of Zika NS2B-NS3pro complexes. (A) One-dimensional ^1^H NMR spectra over -1.2–1.8 ppm of linked (blue) and unlinked (red) Zika NS2B-NS3pro, as well as NS2B (48–74)-NS3pro (green) complexes at a protein concentration of 30 μM. Cyan arrows are used to indicate the very up-field peaks. (B) Superimposition of ^1^H-^15^N HSQC spectra of ^15^N-labeled unlinked (blue) and linked (red) NS2B-NS3pro complexes at a protein concentration of 30 μM. NMR spectra were acquired at 25°C in 10 mM sodium phosphate buffer at pH 6.5. Pink arrows are used to indicate five HSQC peaks for NS2B-Trp61, as well as NS3pro-Trp50, NS3pro-Trp69, NS3pro-Trp83 and NS3pro-Trp89 side chains only observed for the unlinked complex. (C) Far-UV CD spectra of linked (blue) and unlinked (red) Zika NS2B-NS3pro, as well as NS2B (48–74)-NS3pro (green) complexes at a protein concentration of 10 μM, together with that of Dengue-2 NS2B-NS3pro complex previously obtained (black) (ref. 12). (D) Emission spectra of the intrinsic UV fluorescence of linked (blue) and unlinked (red) Zika NS2B-NS3pro, as well as NS2B (48–74)-NS3pro (green) complexes at a protein concentration of 10 μM. (E) Emission spectra of the intrinsic UV fluorescence of unlinked Zika NS2B-NS3pro in 50 mM Tris-HCl buffer at pH 8.5, in the presence of DMSO at different concentrations. (F) Emission spectra of the intrinsic UV fluorescence of unlinked Zika NS2B-NS3pro in 50 mM Tris-HCl buffer at pH 8.5, in the presence of Glycerol as well as DMSO at different concentrations.

First we acquired ^1^H NMR one-dimensional spectra for both linked and unlinked NS2B-NS3pro (Fig 1A). Both spectra have similar up-field peaks, which can only be observed on a well-folded protein with the tight tertiary packing and will disappear even upon a slight disruption to its tight tertiary packing [36]. Fig 1A clearly indicates both linked and unlinked complexes are well folded. An interesting note here is the peaks of linked complex are broader than those of the unlinked complex, which implies the linkage between NS2B and NS3pro introduced additional μs-ms conformational dynamics; this phenomenon was observed for the linked Dengue NS2B-NS3pro [21,30,31]. While this linkage significantly facilitated the crystallization of the linked flavi-viral NS2B-NS3pro complexes [27–29], this linkage significantly broadened NMR signals of linked NS2B-NS3pro complexes [21,30,31]. Thus, high-resolution NMR can be done on the unlinked form of Dengue NS2B-NS3pro which was found to manifest well-dispersed NMR spectra [30,31]. Similarly here, the linked Zika NS2B-NS3pro also had broad NMR signals and consequently only a very small portion of HSQC peaks could be detected (Fig 1B). By contrast, the unlinked complex had sharper 1D up-field peaks (Fig 1A) and a well-dispersed HSQC spectrum (Fig 1B).

We further characterized Zika NS2B-NS3pro complexes with one being labeled and the other unlabeled. As seen in S2A Fig, the ^15^N-labeled NS3pro domain in complex with unlabeled NS2B manifested a well-dispersed HSQC spectrum. While ^15^N-labeled ZIKV NS2B in complex with unlabeled ZIKV NS3pro had a narrowly-dispersed HSQC spectrum with only ~29 peaks detectable (S2B Fig), Dengue NS2B in complex with Dengue NS3pro domain has a well-dispersed HSQC spectrum (S2C Fig) [21,30,31] which unambiguously indicates Dengue NS2B has a tight tertiary packing unlike the Zika one. Therefore, our results suggest that in the Zika NS2B-NS3pro complex, NS2B has a portion of residues undergo μs-ms dynamics which made their NMR peaks too broad to be detectable; while the rest of NS2B is highly disordered and lacks tight tertiary packing, which results in a narrowly-dispersed HSQC spectrum (S2B Fig).

Both linked and unlinked Zika NS2B-NS3pro complexes have far-UV CD spectra with the maximal negative signal at the wavelength of ~201 nm and lacks any positive signal below 200 nm, which is different from that of the unlinked Dengue complex of the same length [21] which has the maximal negative signal at 217 nm and large positive signal at 192 nm (Fig 1C). CD studies provide another piece of evidence that Zika NS2B-NS3pro complexes contain more disordered regions than the Dengue one. We have also collected spectra of intrinsic UV fluorescence from five Trp residues in both linked and unlinked NS2B-NS3pro complexes (Fig 1D), and both complexes have similar spectra with the emission maxima ranging from 344 to 348 nm, very similar to what were observed on the linked NS2B-NS3pro complexes of all four Dengue serotypes (348 nm) [25], which suggests that in Zika NS2B-NS3pro, all five Trp residues are similarly buried as Dengue ones, as Trp residue in the unfolded proteins has an emission maximum wavelength > 352 nm [37].

To screen inhibitors from natural products which are largely insoluble in aqueous buffers, we introduced organic solvents such as dimethyl sulfoxide (DMSO) and glycerol into the activity assay buffers. We assessed their effects on the conformations of Zika NS2B-NS3pro by monitoring intrinsic UV fluorescence. The results indicate that DMSO induced significant changes in the intrinsic UV fluorescence spectra of ZIKV's NS2B-NS3pro (Fig 1E), while glycerol has no significant effect even with concentration up to 20% (Fig 1F).

### Solution conformations of Zika NS2B in different states

**Fig 2:**
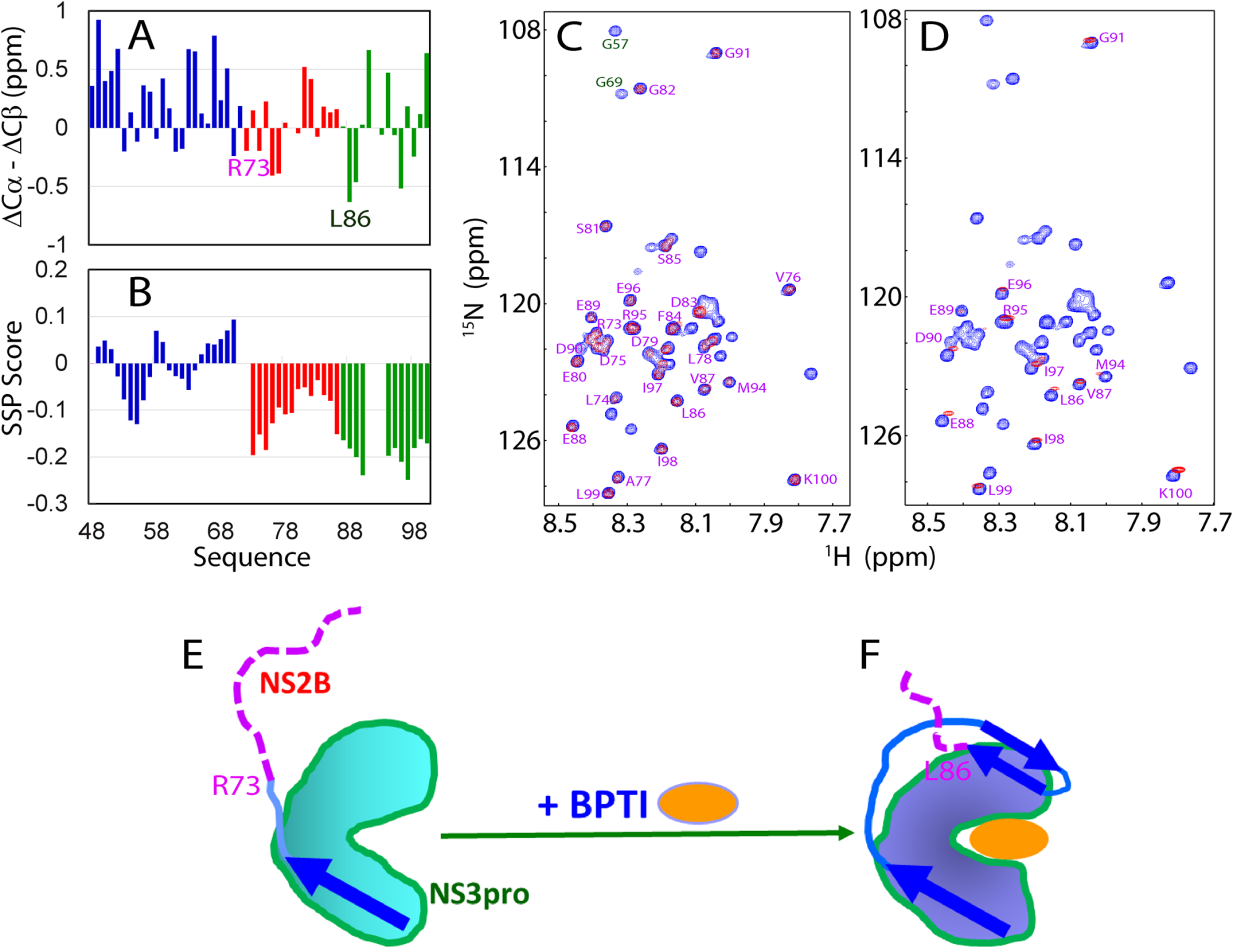
Solution conformations of the Zika NS2B in different states. (A) Residue specific (ΔCα-ΔCβ) chemical shifts of the isolated Zika NS2B. (B) Secondary structure score obtained by analyzing chemical shifts with the SSP program. A score of +1 is for the well-formed helix while a score of -1 for the well-formed extended strand. (C) Superimposition of ^1^H-^15^N HSQC spectra of ^15^N-labeled NS2B alone (blue) and ^15^N-labeled NS2B in complex with unlabeled NS3pro (red). The green is used to highlight two residues Gly57 and Gly69, which could only be detected in the HSQC spectrum of NS2B in the free state. (D) Superimposition of ^15^N-labeled NS2B alone (blue) and ^1^H-^15^N HSQC spectra of ^15^N-labeled NS2B in complex with unlabeled NS3pro in the presence of unlabelled bovine pancreatic trypsin inhibitor (BPTI) at a molar ratio of 1:2 (red). NMR spectra were acquired at 25°C in 10 mM sodium phosphate buffer at pH 6.5. A proposed diagram showing the conformations of NS2B in the open conformation (E) and in the close conformation (F) triggered by complexing with BPTI. Blue arrows are used for indicating β-strands formed over NS2B, while purple dashed lines are for flexible regions of NS2B, whose HSQC peaks could be detected.

As the isolated Zika NS2B (48–100) was soluble in buffers, we acquired triple-resonance experiments HNCACB, CBCA(CO)NH on a double labeled sample, and achieved the sequential assignment for all residues of NS2B (48–100) except for Pro72, Pro92 and Pro93 which have no amide protons. Fig 2A presents its (ΔCα-ΔCβ) chemical shifts, which represent a sensitive indicator of the residual secondary structures in disordered proteins [37,38]. The small absolute values of (ΔCα-ΔCβ) chemical shifts over the whole sequence clearly indicate that the isolated Zika NS2B (48–100) lacks stable secondary structure. We used SSP program [39] to gain quantitative insights into the populations of different secondary structures and found residues Arg73-Lys100 all have small but negative SSP score (Fig 2B) which implies these residues are in extended conformations that are weakly populated [37,39].

Strikingly, superimposing the HSQC spectra of the ^15^N-labeled NS2B in the isolated state against its complex form with unlabeled NS3pro revealed that C-terminal residues Arg73-Lys100 were the detectable HSQC peaks of NS2B in complexed form, while N-terminal residues Ser48-Ser71 were the disappeared HSQC peaks (Fig 2C). Furthermore, we titrated the ^15^N-labeled NS2B in complex with unlabeled NS3pro by adding unlabeled BPTI, which is a tight inhibitor and converts open conformation of flaviviral NS2B-NS3pro complexes to the closed form by both NMR [31] and crystallography [40]. Here, upon adding BPTI, the HSQC peaks of Arg73-Ser85 disappeared and only very weak HSQC peaks of Leu86-Lys100 were detectable (Fig 2D). Previously, we have characterized many binding interactions by NMR and found that the binding-induced disappearance of HSQC peaks was due to intermediate binding affinity (with Kd values between nM and μM), or/and enhancement of μs-ms dynamics triggered by binding and binding interactions between two well-folded proteins [41] and between disordered peptides and a well-folded protein [42]. Our NMR results here indicated that only C-terminal residues of ZIKV NS2B remained disordered both in the complex form (Arg73-Lys100; Fig 2E) and in presence of BPTI (Leu86-Lys100; Fig 2F), and the conformation of these C-terminal residues in ZIKV NS2B in these two forms are similar to the uncomplexed form as their HSQC peaks are superimposable (Fig 2C). Our observations are also supported by two independent observations: the C-terminal residues of NS2B beyond Val87 are completely invisible in the crystal structure of Zika NS2B-NS3pro in the closed conformation [34]; a report on the apo/open-form of Zika NS2B-NS3pro [43] stated “… C-terminus of NS2B (residues 69–87) is largely unseen, highlighting its high intrinsic flexibility.” In sharp contrast, the crystal structure of apo DENV NS2B-NS3pro complex showed the C-terminal loop of NS2B adopts a defined conformation despite some discontinuous electron densities beyond residue 76 [27].

So why do Zika and Dengue NS2B have such radical differences in conformations and dynamics? Examination of NS2B sequences reveals that the Zika NS2B region corresponding to the Dengue NS2B Glu91-Thr96 shows no sequence homology at all (S3 Fig), thus implying sequence variations over this region may at least partly account for the loss of the contacts between the Zika NS2B and NS3pro corresponding to the Dengue NS2B Gln93-Leu95 and NS3pro Leu31-Ser34, thus leading to the high dynamics of Zika NS2B C-half. Thus we cloned and expressed a His-tagged Zika NS2B (48–74) with the C-half deleted. Despite its low expression level and insolubility, we managed to purify a sufficient amount of NS2B (48–74) for refolding with NS3pro with the same protocol described above. Interestingly, NS2B (48–74) is sufficient to form a soluble complex with NS3pro (S1E Fig). However, its NMR peaks are broader than those of the linked NS2B-NS3pro (Fig 1A), which is likely due to μs-ms conformational dynamics or/and dynamic aggregation. On the other hand, its intrinsic UV fluorescence spectrum indicates that its four Trp residues are similarly buried as linked and unlinked Zika complexes with the full-length soluble domain of NS2B (Fig 1D). Most interestingly, this complex with the C-half of NS2B deleted contains less amount of disordered region than both linked and unlinked Zika complexes with the full-length NS2B, as seen by its CD spectrum having maximal negative signal shifted to 207 nm and has positive elliptical signal at 191 nm (Fig 1C), Despite being less disordered, this complex (with the later C-half of NS2B deleted) showed no detectable enzymatic activity even with the protease concentration up to 20 μM, which suggests this complex is enzymatically inactive. Previously, we generated a truncated Dengue NS2B with the same C-half deleted but the shorter NS2B is unable to form a soluble complex with its NS3pro [21]. However, we also generated another truncated Dengue NS2B with only residues 77–84 deleted, which was designated as NS2B (48–100; Δ77–84). Interestingly, NS2B (48–100; Δ77–84) was able to form a soluble but inactive complex with its NS3pro, which appeared to be highly disordered as reflected by its CD spectrum, and highly dynamic as judged by its NMR spectrum [21].

Together with recent reports on the crystal structures of Zika NS2B-NS3pro complexes in both open and closed conformations [34,43], our current results reveal that in solution the NS2B residues over Arg73-Lys100 are highly disordered in the open conformation. However, upon conversion into closed conformation such as triggered by BPTI binding, the NS2B residues Arg73-Ser85 become further bound to the NS3pro domain. On the other hand, our results suggest that despite being intrinsically disordered [44], the C-half of Zika NS2B is absolutely required for implementing the catalytic actions, thus implying that the closed conformation might be enzymatically-active, which was also previously speculated [27–30,34,40]. Additionally, being disordered for the NS2B C-half might have other functional advantages offered by intrinsically disordered proteins [44], such as to allow the formation of dynamic replication complex observed in HCV replication [45,46].

### Characterization of the enzymatic catalysis

We have extensively characterized the catalytic properties of both linked and unlinked Zika NS2B-NS3pro complexes in different buffer conditions. To allow comparison with the previously published results on the (Gly)_4_-Ser-(Gly)_4_ linked NS2B-NS3 proteases of all four Dengue serotypes [26], we have selected the same three substrates. These substrates, namely Bz-nKRR-AMC, Boc-GRR-AMC and Boc-GKR-AMC, were extensively used for characterization of other flaviviral NS2B-NS3pro. Moreover, we have used the same buffer as previously published [26], for the measurement of proteolytic activities. Furthermore, the effects of salts and organic solvents on the Zika protease activity were assessed. Interestingly, as shown in S4A Fig, the Zika NS2B-NS3pro efficiently cleaved Bz-nKRR-AMC, but unexpectedly showed weak activity on other two substrates. These results imply that besides requiring dibasic residues at the P1 and P2 sites (which is characteristic of the flaviviral NS3 proteases), Zika NS2B-NS3pro appears to have a higher need for a basic residue at P3 site than Dengue complexes [26]; as our current focus was not on profiling substrate specificity, we did not test this hypothesis on more substrates but instead measured all kinetic constants of Zika complexes with Bz-nKRR-AMC.

We found enzymatic activities of both linked and unlinked ZIKV complexes showed similar dependence on the pH values with the optimal pH at ~9.5 (S4B Fig), which were similar to other flaviviral NS2B-NS3pro linked by (Gly_4_)-Ser-(Gly_4_) [23–26], different from Dengue-2 NS2B-NS3pro, for which the optimal pH significantly switched from basic pH to neutral pH upon separating NS2B from NS3pro [30]. Secondly, like other flaviviral NS2B-NS3pro, the catalytic activity of Zika NS2B-NS3pro also reduced significantly upon increasing the concentrations of NaCl (S4C Fig). In the presence of 150 mM NaCl, the catalytic activity is only 13.6% and 8.2% of the linked and unlinked enzymes respectively in 50 mM Tris buffer at pH 8.5. Thirdly, glycerol concentrations also largely affect the enzymatic activity. The linked Zika complex showed the highest activity in the presence of 30% glycerol while the unlinked had the highest activity in the presence of 20% glycerol (S4D Fig).

Hence, we measured the kinetic parameters for the unlinked complex in the same buffers but with varying concentrations of organic solvents (S4E Fig) and the results were presented in Table 1. The linked and unlinked Zika complexes only have slightly differences for their kinetic constants in the buffer without any organic solvent. Recently, the kinetic constants were just published for a Zika NS2B-NS3pro which has NS2B (49–95) and NS3 (1–170) also linked by the same (Gly)_4_-Ser-(Gly)_4_ linker [34]. It has a Km of 18.3 μM and kcat of 44.6 s^-1^ respectively. While the kcat values are almost identical, the Km value is ~2.4-fold less than our current one. This small difference is likely due to: 1) the difference of substrate as Bz-nKKR-AMC was used in the study [34]; or/and 2) the slight differences of NS2B and NS3pro lengths, 3) or/and the difference of the buffers: our buffer is 50 mM Tris pH 8.5 while the buffer in the publication [34] is only 10 mM Tris pH 8.5 with 20% glycerol and 1 mM CHAPS. It is extensively demonstrated that high salt concentrations has decreased, while glycerol at low concentrations has increased the activity of the flaviviral proteases [21–27]. Indeed, we measured the activity of our linked Zika protease in 10 mM Tris pH 8.5, and the catalytic activity is 1.36-time higher.

**Table 1:** Kinetic parameters of Zika NS2B-NS3pro in different buffers.

	Km μM)	kcat (s^-1^)	kcat/Km (M^-1^s^-1^)
**Linked complex** (50 mM Tris buffer pH 8.5)	43.4 ± 2.0	48.9 ± 0.4	1,128,681 ± 56,053
**Unlinked complex** (50 mM Tris buffer pH 8.5)	60.2 ± 0.7	93.1 ± 1.1	1,545,992 ± 20,045
**Unlinked complex** (50 mM Tris buffer, pH 8.5 + 25% DMSO)	134.6 ± 10.7	103.5 ± 9.3	765,712 ± 58,361
**Unlinked complex** (50 mM Tris buffer pH 8.5 + 20% Glycerol)	75.9 ± 7.2	171.5 ± 1.7	2,279,450 ± 214,520
**Unlinked complex** (50 mM Tris buffer pH 8.5 + 20% Glycerol + 1% DMSO)	85.1 ± 4.8	126.5 ± 2.2	1,482,086 ± 62810

### Screening and characterization of natural product inhibitors

Due to the urgency to fight ZIKV infection, we attempted to screen the inhibitors of Zika NS2B-NS3pro from natural products rich in edible plants. To better reflect the situation *in vivo*, here we selected the unlinked Zika NS2B-NS3pro complex for screening. However as most natural products are largely insoluble in aqueous buffers, we have assessed effects of DMSO and glycerol on the conformations (Fig 1E and 1F) as well as on catalytic parameters (Table 1). Consequently, 50 mM Tris buffer at pH 8.5 + 20% Glycerol was utilized as the screening buffer as it could dissolve all natural products in the present study but has no significant effect on the conformation of Zika NS2B-NS3pro (Fig 1F).

**Fig 3:**
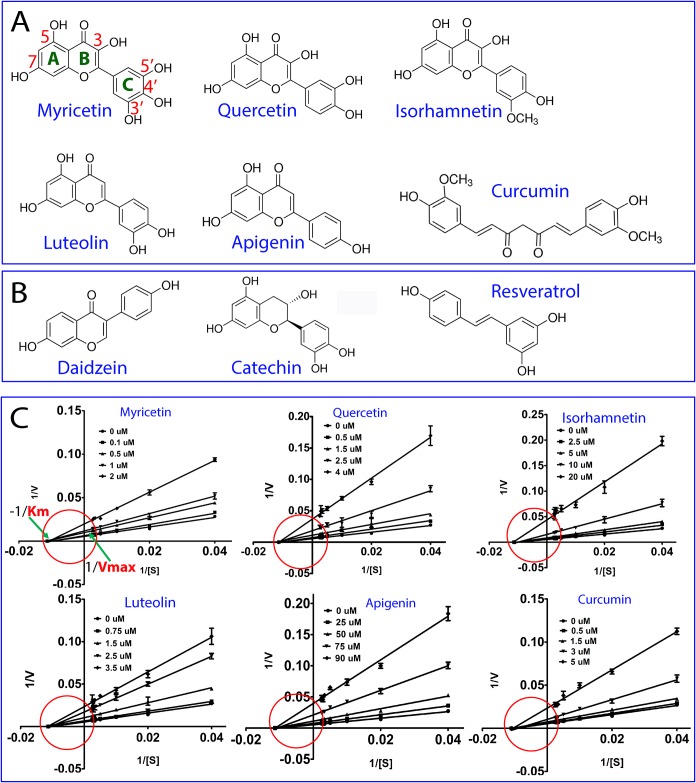
Identification of natural products inhibiting Zika NS2B-NS3pro. (A) Chemical structures of six natural products identified to inhibit Zika NS2B-NS3pro. (B) Chemical structures of three natural products identified to have no detectable inhibitory activity on Zika NS2B-NS3pro. (C) Lineweaver-Burk plots for determining inhibitory constants (*K*i) of six natural products on Zika NS2B-NS3pro. [S] is the substrate concentration; v is the initial reaction rate. The curves were generated by the program GraphPad Prism 7.0. The red circles are used to indicate that the inhibition is non-competitive, characteristic of the same Km but varying Vmax values in the presence of inhibitors at different concentrations.

Remarkably, we have identified six compounds to have significant inhibitory effects, which belong to flavonoid and natural phenol (Fig 3A). Subsequently, we have determined their values of IC_50_ (S5 Fig and Table 2); and inhibitory constant *K*i (Fig 3C; Table 2). Noticeably, despite sharing the same scaffold, five flavonoids have very distinctive inhibitory effects, with Myricetin being the highest (IC50 of 1.26 μM and *K*i of 0.77 μM) and Apigenin the weakest (IC50 of 56.32 μM and *K*i of 34.02 μM). On the contrary, no inhibitory activity was detected even at a compound concentration up to 500 μM for Daidzein, an isoflavone; as well as Catechine that has a chemical structure identical to that of Quercetin, except that the C-ring of Catechin is a dihydropyran heterocycle (Fig 3B). Noticeably, Isorhamnetin, also called 3’-Methylquercetin, has a much weaker inhibitory activity (IC50 of 15.46 μM and *K*i of 6.22 μM) than Quercetin (IC50 of 2.42 μM and *K*i of 1.12 μM), although Isorhamnetin is a derivative of Quercetin only with proton of the hydroxyl group at 3’ position of phenyl ring replaced by a methyl group (Fig 3A). By contrast, Quercetin and Luteolin have almost the same inhibitory activity (Table 2), although a hydroxyl group at 3 position of benzopyran ring is absent in Luteolin (Fig 3A). Furthermore, a significant inhibitory effect (IC50 of 3.45 μM and *K*i of 2.61 μM) was detected for Curcumin, a natural phenol with two aromatic rings linked by heptadiene group (Fig 3A), while no inhibitory effect was detected for Resveratrol, also a natural phenol with two aromatic rings but linked by unsaturated ethene group (Fig 3B). These results suggest that these natural products inhibit Zika NS2B-NS3pro specifically.

**Table 2:** Inhibitory parameters of six natural products on Zika NS2B-NS3pro.

	Inhibition at 500 μM	IC_50_ (μM)	*K*_i_ (μM)
**Myricetin**	Yes	1.3 ± 0.1	0.8 ± 0.1
**Quercetin**	Yes	2.4 ± 0.2	1.1 ± 0.1
**Luteolin**	Yes	2.7 ± 0.3	1.4 ± 0.1
**Isorhamnetin**	Yes	15.5 ± 0.7	6.2 ± 0.4
**Apigenin**	Yes	56.3 ± 0.9	34.0 ± 2.4
**Curcumin**	Yes	3.5 ± 0.2	2.6 ± 0.2
**Catechin**	No	NA	NA
**Daidzein**	No	NA	NA
**Resveratrol**	No	NA	NA

Another significant finding is that the six compounds inhibit Zika NS2B-NS3pro by changing Vmax but not Km (Fig 3C). This indicates that these compounds inhibit Zika NS2B-NS3pro in a non-competitive mode. In other words, six compounds are most likely to allosterically inhibit Zika NS2B-NS3pro with their binding sites having no overlap with that for the substrate. Indeed, previously Myricetin and Quercetin have been characterized to allosterically inhibit Dengue-2 NS2B-NS3pro with *K*i values of 4.7 and 20.7 μM respectively, which, however, are much weaker than those for Zika NS2B-NS3pro here. Unfortunately, NMR spectroscopy cannot be utilized to investigate the interaction between those compounds and Zika NS2B-NS3pro as the presence of 20% glycerol significantly increased the rotational tumbling time of the protein which made NMR peaks too broad for detection.

### Complexes between Zika NS2B-NS3pro and six active compounds

**Fig 4:**
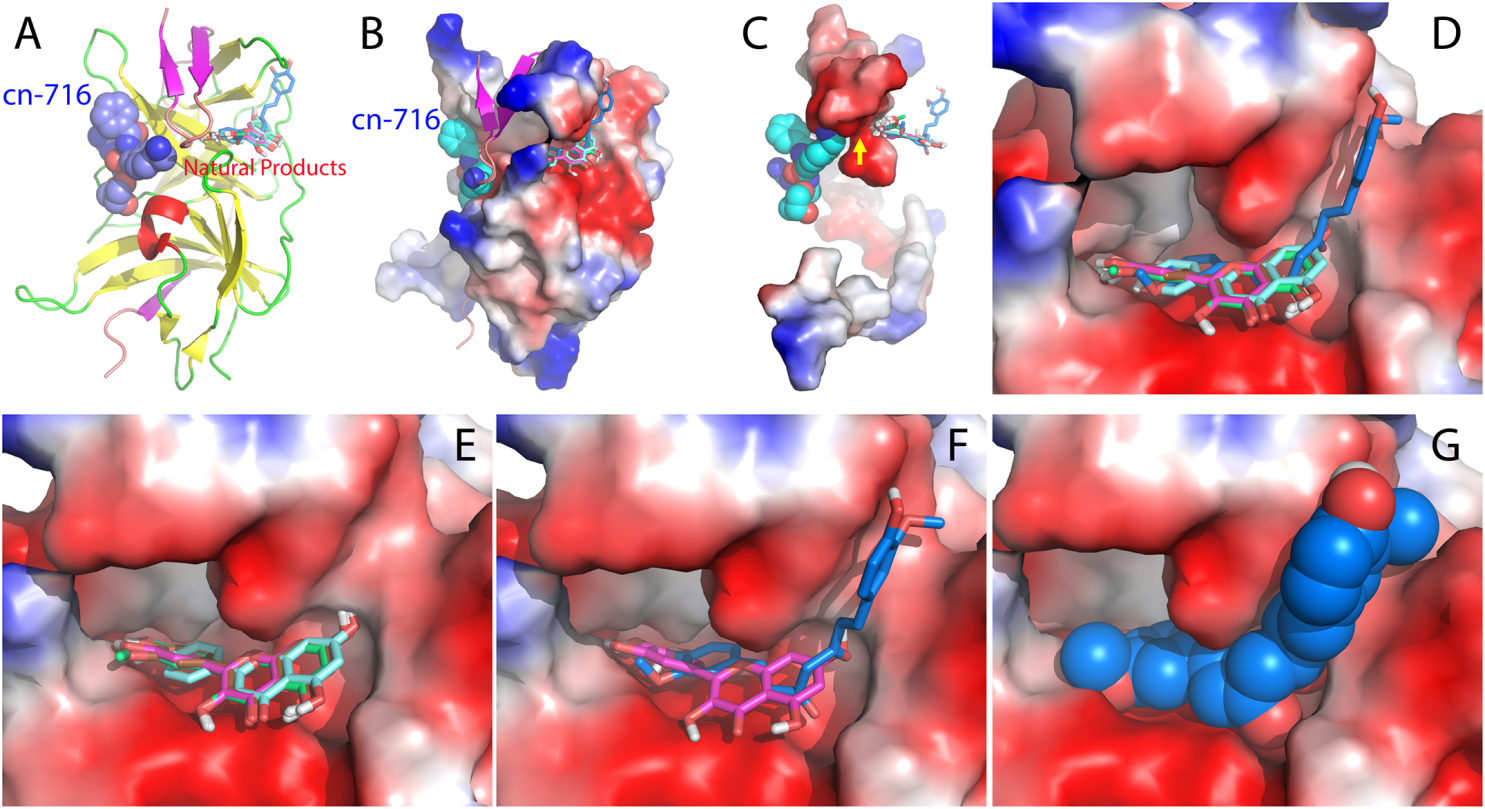
Binding pockets of six natural products on Zika NS2B-NS3pro. (A) The crystal structure (PDB code of 5LC0) of Zika NS2B-NS3pro determined with an active site inhibitor cn-716 (in spheres); to which six natural products (in sticks) were docked. Green, red and yellow are used respectively to color loop, helix and β-strand of NS3pro domain, while brown, cyan and purple are respectively for loop, helix and β-strand of NS2B. (B) Zika NS2B-NS3pro in complex with cn-716 and six natural products in which NS2B is displayed in ribbon and NS3pro domain in the electrostatic potential surface. (C) cn-716 and six natural products in complex with NS2B in the electrostatic potential surface. For clarity, only NS2B is displayed in the electrostatic potential surface. Yellow arrow is used to indicate the NS2B region which contacts cn-716 on the one side and the natural product inhibitors on the other. Expanded binding pockets of Zika NS2B-NS3pro in complex with all six compounds (D); with five flavonoids (E); with Myricetin and Curcumin (F); and only with Curcumin in spheres (G).

To facilitate a better understanding of the experimental results and elucidate structure-activity relationship of the compounds, we used AutoDock software [47] to dock six small molecules to the crystal structure (5LC0) [34] of ZIKV NS2B-NS3pro with the substrate-derived inhibitor cn-716 removed. Strikingly, all six compounds bind to the pockets on the back of the active site of Zika NS2B-NS3pro (Fig 4A), similar to flavonoids binding to Dengue-2 NS2B-NS3pro such as Myricetin and Quercetin [32]. Interestingly in the complexes, the short β-sheet formed by NS2B residue Leu74-Leu78 and Asp83-Leu86 has direct contacts with the active site inhibitor cn-716 on one side, and with the six compounds on another side (Fig 4B and 4C). As such the pocket for binding six compounds is constituted by the surfaces provided by both Zika NS2B and NS3pro (Fig 4D and 4E), whose inner surfaces are relatively polar and negatively charged. Interestingly, five flavonoids are highly superimposable in the pocket with the phenyl ring contacting the inner wall formed by NS2B, and with the benzopyran ring located in a pocket provided by NS3pro (Fig 4E). Amazingly, although one phenyl ring of Curcumin also occupies the same pocket like flavonoids, another phenyl ring has established the binding to a new pocket of NS3pro, which is not observed for five flavonoids (Fig 4F and 4G).

Previously a similar pocket has been extensively identified on the Dengue-2 NS2B-NS3pro structure 3U1I [40] to bind flavonoids including Myricetin and Quercetin [32,48]. However, the binding affinities of Myricetin and Quercetin to bind Dengue NS2B-NS3pro are much weaker than those for Zika one. As seen in S6 Fig, although the overall RMSD of all heavy atoms is only 0.62 Å between the crystal structures of Zika [34] and Dengue-2 [32] NS2B-NS3pro complexes, which are both bound with active-site inhibitors, the binding pockets have slightly-different local geometries but very distinctive electrostatic properties: the Zika pocket is more polar and negatively charged, while the Dengue-2 pocket is less polar and positively charged (S6 Fig).

**Fig 5:**
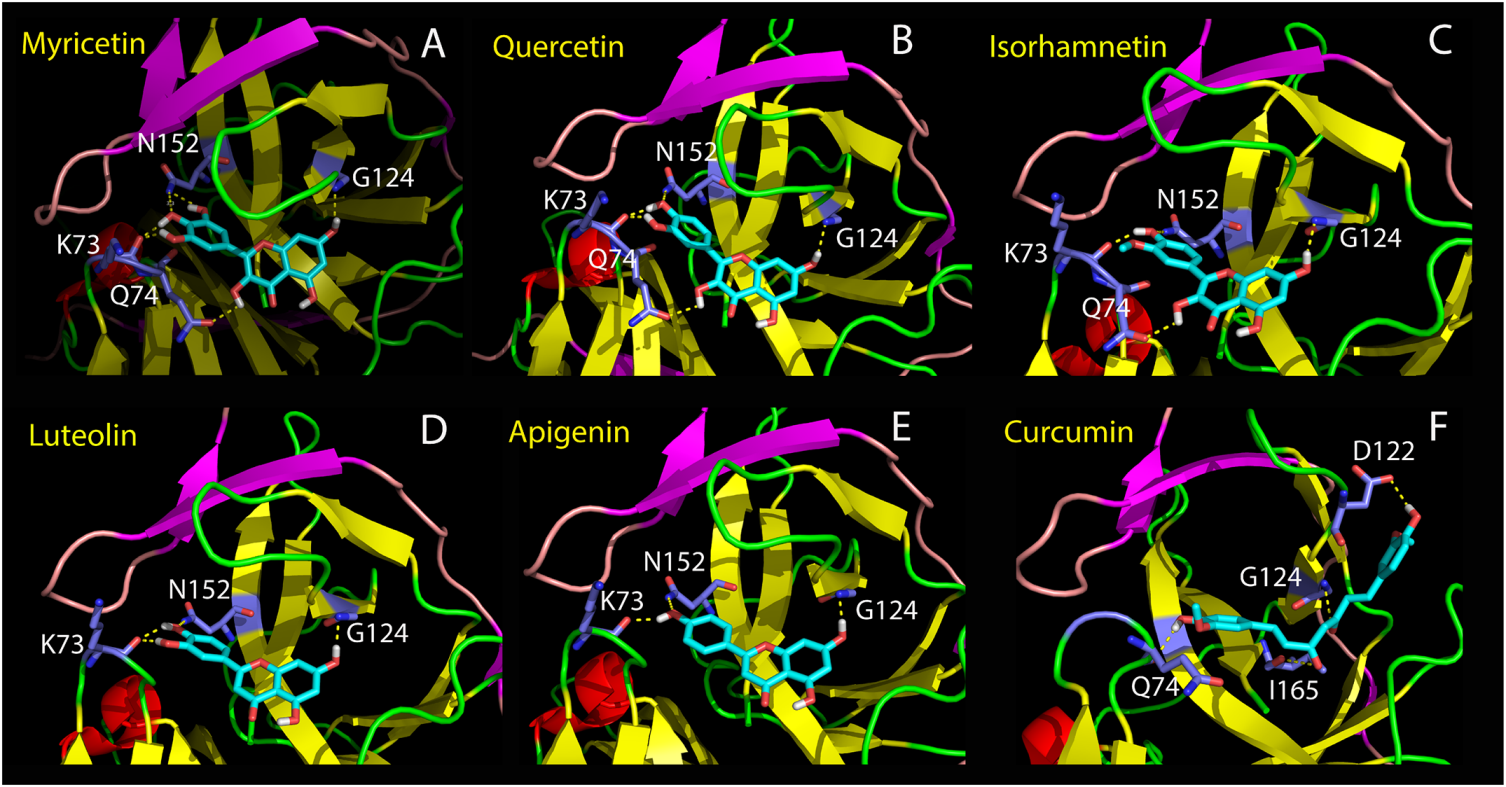
Hydrogen bond networks between Zika NS2B-NS3pro and six natural products. Hydrogen bonds (in dashed yellow lines) formed between atoms of Zika NS2B-NS3pro and Myricetin (A); Quercetin (B); Isorhamnetin (C); Luteolin (D); Apigenin (E) and Curcumin (F).

We further analyzed the hydrogen bond networks formed between Zika NS2B-NS3pro and six compounds. Interestingly, Myricetin, the strongest inhibitor, establishes six hydrogen bonds with four Zika NS3pro residues (Fig 5A). Protons of 3’, 4’-hydroxyl groups on phenyl ring form two hydrogen bonds with the backbone oxygen atoms of Lys73 of the Zika NS3pro domain, while oxygen atoms of 4’, 5’-hydroxyl groups on phenyl ring establishes two hydrogen bonds with the side chain NH atom of Asn152. Furthermore, proton of 3-hydroxyl group on the benzopyran ring forms a hydrogen bond with the side chain oxygen atom of Gln74, while proton of 7-hydroxyl group on benzopyran ring forms a hydrogen bond with the backbone nitrogen atom of Gly124.

Due to the absence of 5’-hydroxyl group on phenyl ring in Quercetin, one hydrogen bond is missing between oxygen atom of 5’-hydroxyl groups and the side chain NH atom of Asn152 (Fig 5B), which may at least partly account for its slightly weak affinity than that of Myricetin. Strikingly, due to the replacement of proton of 3’-hydroxyl group of Quercetin by a methyl group in Isorhamnetin, the hydrogen bond between proton of 3’-hydroxyl group and backbone oxygen of Lys73 is lost (Fig 5C). This hydrogen bond appears to significantly contribute to the inhibitory activity as the inhibitory constant *K*i of Isorhamnetin increases by ~6 times as compared to Quercetin (Table 2).

On the other hand, Luteolin establishes the same hydrogen bond network with Zika NS2B-NS3pro residues as Quercetin except for the loss of the hydrogen bond with Gln74 due to the absence of 3-hydroxyl group in Luteolin (Fig 5D). However, this hydrogen bond appears to be not very important as only a very slight reduction of the inhibitory activity was observed for Luteolin as compared to Quercetin (Table 2). However, the further absence of 3’-hydroxyl group on phenyl ring in Apigenin results in loss of a hydrogen bond between 3’-hydroxyl group on phenyl ring and backbone oxygen atom of Lys73 (Fig 5E). This hydrogen bond appears to be critical for its inhibitory activity as Apigenin has a Ki increased by ~25 times as compared to Luteolin (Table 2).

Amazingly, Resveratrol has no detectable inhibitory activity but Curcumin shows strong inhibitory activity comparable to Quercetin. In fact, Resveratrol and Curcumin have similar structures but the linker between two phenyl rings of Resveratrol is 5-carbon shorter than that of Curcumin. The complex model between Zika NS2B-NS3pro and Curcumin (Fig 5F) provides an explanation to the experimental result. With a longer linker, one phenyl ring of Curcumin occupies the pocket identical to five flavonoids with the formation of hydrogen bonds with Gln74 and Gly124, while another phenyl ring has additional contacts with a new pocket with unique hydrogen bonds with Asp122 and Ile165. As such, although Curcumin and Isorhamnetin have the same 3’-methoxy and 4’-hydroxyl groups on the phenyl rings, likely by having bivalent binding sites, Curcumin gains an inhibitory affinity which is much higher than that of Isorhamnetin (Table 2). A high affinity, which is achieved by establishing bivalent or multivalent binding sites, has been extensively found, such as on bivalent thrombin-inhibitor interactions [49].

## Discussion

Knowledge of catalysis, structures and dynamics of all structural states is beneficial for design of inhibitors with high affinity and specificity towards enzymes including viral proteases [49–53]. This knowledge is particularly relevant to the flaviviral NS2B-NS3pro complexes as it is proposed that their catalytic activities require a transition from the open (inactive) to closed (active) conformation [21,28,29,40]. Interestingly, regardless of being in the open or closed conformations, NS3pro domains of different flaviviral NS2B-NS3pro complexes universally adopt the same chymotrypsin fold. By contrast, while the N-half of NS2B assumes a similar β-strand packed to the NS3pro domain in both open and closed conformations, the C-half of NS2B shows a significant structural diversity in different structures determined so far. In the closed conformation, NS2B structures of flaviviral NS2B-NS3pro complexes show a similar a short β-hairpin formation over the C-half of NS2B and is tightly bound to the NS3pro chymotrypsin fold (Fig 4A). However in the open conformation, the structural properties of the NS2B C-half have been shown to be quite diverse. For the well-studied Dengue-2 NS2B-NS3pro in the open conformation, most NS2B residues are tightly packed with the NS3pro domain as revealed by the crystal structure [27], and evident from its well-dispersed HSQC spectrum (S2C Fig) reconstructed from a previous report [30].

In the present study, we first constructed and characterized the Zika NS2B-NS3pro complex with NS2B and NS3pro linked by an artificial (Gly)_4_-(Ser)-(Gly)_4_ sequence which has been found to dramatically facilitate the crystallization of flaviviral NS2B-NS3pro complexes [27,34,40,43]. Despite slight differences in sequence length, the catalytic parameters (Table 1) of our linked Zika NS2B-NS3pro complex have no significant difference from those recently published [34]. Unfortunately, as previously observed on Dengue-2 NS2B-NS3pro complexes [21,30,43], our linked Zika complex also underwent significant μs-ms dynamics, thus making its NMR signals too broad to be detected (Fig 1A and 1B). As a consequence, we devoted efforts to generate and characterize an unlinked Zika NS2B-NS3pro complex by using a protocol we previously established for the Dengue-2 NS2B-NS3pro complex [21]. This approach is also required for the selective isotope-labeling of Zika NS2B or NS3pro for high-resolution NMR studies. Indeed, despite showing no significant difference of catalytic properties from the linked one (Table 1), the unlinked Zika NS2B-NS3pro complex suddenly manifested a well-dispersed HSQC spectrum of the ^15^N-labeled NS3pro domain in complex with unlabeled NS2B with sharper NMR peaks (Fig 1A and 1B), which are consistent with previous NMR results on the unlinked Dengue complexes [21,30,31]. Most importantly, this allowed us to selectively study the ^15^N-labeled NS2B in complex with unlabeled NS3pro. The results revealed that the Zika NS2B-NS3pro complex, the C-terminal residues Arg73-Lys100 of NS2B remain highly disordered unlike the Dengue-2 NS2B-NS3pro complex in the open conformation. Binding to BPTI appeared to trigger the conversion of Zika NS2B-NS3pro complex into the closed conformation, in which the NS2B C-terminal residues Arg73-Ser85 become further bound to the NS3pro domain. The intrinsic dynamics of the Zika NS2B C-half might be due to the significant sequence variations over NS2B residues 91–96 (S3 Fig). Strikingly, this unique property for Zika NS2B-NS3pro is not only observed in solution by our NMR investigation, but has been recently shown by the crystal structure of the apo/open-form of Zika NS2B-NS3pro [43]. In the future, it is of significant interest to explore what is the functional consequence of this unique property. One possibility might be that with the intrinsically disordered NS2B C-half [44], the Zika NS2B-NS3pro is more susceptible to the allosteric regulation [50–52].

Although many adults infected with ZIKV will have only mild or even no detectable symptoms, the ZIKV can be transmitted from a pregnant woman to her fetus, thus leading to birth defects such as microcephaly. This imposed a great challenge and urgency to fight ZIKV. Therefore we attempted to screen inhibitors from natural products rich in edible plants for the unlinked Zika NS2B-NS3pro, which represents a more realistic form *in vivo*. Remarkably, we identified five flavonoids and one natural phenol to specifically inhibit Zika NS2B-NS3pro with the strongest Myricertin having a *K*i of 770 nM. Recently, Lim et. al. has reported a Ki value of 8.9 uM for Myricitin [54], the difference might be due to 1) the linked Zika NS2B-NS3pro which they have used for their enzyme inhibition assay whereas we have used an unlinked enzyme and 2) the pH of the assay buffer they used is pH 7 whereas we have used pH 8.5. Furthermore, analysis of structures and inhibitory activities reveals that for flavonoids, the presence of benzopyran ring and the position to connect to phenyl ring are critical for the inhibitory activity, as evident from the results that Daidzein and Catechin were inactive on inhibiting Zika NS2B-NS3pro. Furthermore, the number of hydroxyl groups on phenyl ring is a key determinant for the inhibitory activity. Similar observation has been reported in a recent study where a number of polyphenols has been studied for inhibitory activity against linked Zika NS2B-NS3pro [54]. Interestingly, our current molecular docking reveals that these compounds bind to a pocket on the back of the active site of the Zika NS2B-NS3pro complex. While five flavonoids bind very similarly to the pocket constituted by both NS2B and NS3pro, Curcumin can have an additional binding site outside of this pocket. Amazingly, the pocket we observed on the Zika NS2B-NS3pro to bind five flavonoids is very similar to that previously observed on the Dengue NS2B-NS3pro [32,48], but the additional binding site for Curcumin represents a novel one. Therefore, Zika NS2B-NS3pro appears to be highly susceptible to allosteric inhibitions, as previously found on other flaviviral NS2B-NS3pro complexes [24,32,48,53]. Noticeably, probably due to slight variations of the local geometry or/and significant difference in electrostatic potentials of the allosteric pockets of Zika and Dengue complexes, the same flavonoids inhibit the Zika NS2B-NS3pro complex with higher activity [32].

Five flavonoids and curcumin are rich in various edible plants including caper, tea, red onion, celery, broccoli, green pepper and turmeric. Additionally, they have been previously demonstrated to have beneficial effects on human health and thus extensively used as supplements [32,55–57]. As such, our finding might help the public to fight against ZIKV infection immediately. In the further, it is also of both fundamental and therapeutic interests to characterize the allosteric mechanisms by which these natural products inhibit Zika NS2B-NS3pro by both experimental and computational approaches [53–60]. As these natural product inhibitors fundamentally differ from the currently-known active site inhibitors in both inhibitory mode and chemical scaffold [34,43], our finding may also provide a promising foundation for further development of novel allosteric inhibitors of higher affinity and specificity to fight ZIKV infection.

## Material and methods

### Plasmid construction

The identified genes encoding NS3pro (1–185) and NS2B (1–130) from the Asian Zika strain 8375 (GenBank ID: KU501217.1) were optimized and synthesized by GenScript (Piscataway, NJ). With designed primers, the genes by GenScript were used as templates for amplifying DNA fragments encoding the isolated NS3 (14–185) and NS2B (48–100) with the transmembrane regions removed, as well as NS2B (48–74) with the C-region further deleted. Furthermore, DNA fragments were designed for linking NS2B (48–100) to NS3 (14–185) by (Gly)_4_-Ser-(Gly)_4_ using overlap PCR as previously described (14). Amplified DNA fragments were subsequently cloned into His-tagged pET28a vector (Novagen). DNA sequences of all constructs were verified by automated DNA sequencing.

### Protein expression and purification

All pET28a vectors containing different Zika genes were transformed into *Escherichia coli* BL21 (DE3) Star cell (Thermo Fisher Scientific), which was cultured in Luria-Bertani broth containing 25 μg/ml kanamycin at 37°C until the A600 reached 0.6. Subsequently, protein expressions of different constructs were induced with different conditions. To obtain soluble form of the linked NS2B-NS3pro complex, protein expression was induced with 0.2 mM isopropyl β-D-thiogalactopyranoside (IPTG) overnight at 18°C. However, the expression level of the soluble form was low and consequently the linked complex was also prompted into inclusion body with induction of 1 mM IPTG for 4 hours at 37°C, which yielded to a much higher expression level. For isolated NS2B and NS3, protein expressions were induced with 1 mM IPTG for 4 hours at 37°C. For the linked NS2B-NS3pro complex, the soluble form was purified by Ni^2+^-affinity chromatography under native condition.

Subsequently, the His-tag was removed by the cleavage with thrombin-agarose beads from Thrombin CleanCleave^™^ Kit (Sigma-aldrich, St. Louis, MO), followed by binding to an excess amount of Ni-NTA beads to remove His-tag and uncleaved fusion protein, as well as a final FPLC purification on a gel filtration column (HiLoad 16/60 Superdex 200). To obtain the linked complex in inclusion body, the cell pellets were re-suspended in cold PBS buffer at pH 7.4, containing 10 mM β-mercaptoethanol and 8 M urea and the supernatant containing the recombinant proteins were purified by Ni-NTA affinity column under denaturing condition. Eluted fractions were subjected to dialysis against PBS buffer (pH 7.4) with 10 mM β-mercaptoethanol at 4°C overnight to allow the refolding of the complex. The refolded complex was subjected to the cleavage of His-tag and final FPLC purification as described above.

The isolated NS2B (48–74) and NS3 (14–185) were purified by Ni-NTA affinity column under denaturing condition, while NS2B (48–100) was purified under native condition. Subsequently, the purified NS2B (48–100)/NS2B (48–74) and NS3 (14–185) were mixed up with an excess amount of NS3. The mixture was subjected to refolding by dialysis against PBS buffer (pH 7.4) with 10 mM β-mercaptoethanol at 4°C overnight as we previously established for Dengue NS2B-NS3pro complex (12). The refolded complex was subjected to the cleavage of His-tag and further FPLC purification as described above.

The generation of the isotope-labeled proteins for NMR studies followed a similar procedure except that the bacteria were grown in M9 medium with the addition of (^15^NH_4_)_2_SO_4_ for ^15^N labeling and (^15^NH_4_)_2_SO_4_/[^13^C]-glucose for double labeling [21]. All recombinant protease samples were checked by Tris-Glycine SDS PAGE. However, for this SDS-PAGE system, small peptides diffuse significantly and thus usually cannot be seen. Therefore, reverse-phase (RP) high pressure liquid chromatography (HPLC) with an analytic C8 column was used to check the presence of the isolated NS2B peptides with a molecular weights less than 6 kDa. Molecular weight verification and protein sequencing were conducted with time-of-flight-mass spectrometer (Applied Biosystems). Protein concentration was determined by the UV spectroscopic method with 8 M urea [21].

### Fluorescence and CD experiments

Intrinsic UV fluorescence spectra were measured with a Cary Eclipse fluorescence spectrophotometer as we previously described [37] with the excitation wavelength at 280 nm. Circular dichroism (CD) experiments were performed on a Jasco J-1500 spectropolarimeter and data from five independent scans were added and averaged [21]. To assess the effects of DMSO and glycerol on the conformation of Zika NS2B-NS3pro, we monitored the change of its intrinsic UV fluorescence instead of circular dichroism (CD), because organic solvents were found to provoke very high non-specific noises.

### NMR experiments

All NMR experiments were acquired on an 800 MHz Bruker Avance spectrometer equipped with pulse field gradient units as described previously [21]. To achieve sequential assignment, ^15^N-/^13^C-double labeled Zika NS2B sample was prepared at a protein concentration of 200 μM in 10 mM phosphate buffer. A pair of triple-resonance experiments HNCACB, CBCA(CO)NH were acquired [21]. To investigate the binding interaction between Zika NS2B-NS3pro and BPTI, HSQC spectra of Zika NS2B-NS3pro only with NS2B ^15^N-labeled were acquired in the absence and in the presence of BPTI (Sigma-Aldrich) at different ratios.

### Enzymatic activity and kinetics

To allow comparison with the NS2B-NS3pro complexes of four Dengue serotypes (17), we selected three fluorophore-tagged substrates previously used (17): namely Bz-Nle-Lys-Arg-Arg-AMC, Boc-Gly-Arg-Arg-AMC and Boc-Gly-Lys-Arg-AMC (Bachem AG, Bubendorf), which were dissolved in dimethyl sulfoxide for preparing stock solutions (100 mM). All enzymatic experiments were performed in triplicate and data are presented as mean ± SD, while IC_50_, Km and *K*i were obtained by fitting with GraphPad Prism 7.0 [61].

The pH dependence was measured with a protease concentration of 50 nM and substrate (Bz-nKRR-AMC) concentration of 250 μM at 0.5 pH intervals using the following buffers: 50 mM citrate-phosphate buffer for pH 4–5, 50 mM phosphate buffer for pH 5.5–8, 50 mM Tris-HCl buffer for pH 8.5–9.5, and 50 mM Na-bicarbonate buffer for pH 10–10.5.

For steady state kinetics, we used the exactly the same buffer as a previous one on profiling substrate specificity for the NS2B-NS3pro complexes of all four Dengue serotypes 17): 50 mM Tris-HCl at pH 8.5. To screen natural product inhibitors of Zika NS2B-NS3pro, we also measured the Km values of Zika NS2B-NS3pro in the presence of DMSO and glycerol which allow the solubilization of these compounds in the assay buffer. Briefly, Zika protease at 50 nM was incubated with substrates ranging from 10 to 1000 μM in 100 μl assay buffer at 37°C. Progression of enzymatic reaction was monitored as an increase in fluorescence at λ_ex_ of 380 nm and λ_em_ of 450 nm. Fluorescence intensity is reported in arbitrary units. Initial fluorescence or absorbance velocities (relative fluorescence units per minute or relative absorbance units per minute) were converted to MS^-1^ from a standard ACMC calibration curve. Subsequently, the curves were fitted to the Michaelis-Menten equation by nonlinear regression.

### Inhibitor screening and determination of inhibitory parameters

All natural products were purchased from Sigma-Aldrich, which are all HPLC purified. After optimization of buffer conditions, here, we selected 50 mM Tris-HCl buffer at pH 8.5 in the presence of 20% glycerol which could dissolve all natural products. Briefly, for the initial screening, the Zika protease at 50 nM was preincubated for 30 min with different compounds at final concentrations of 5 and 500 μM dissolved in 1 μl DMSO, followed by adding Bz-nKRR-AMC to 250 μM to initiate reaction. Only the compounds showing significant inhibitions at both concentrations were subjected to further determination of IC_50_ and *K*i.

For IC_50_ determination, the Zika protease at 50 nM was preincubated at 37°C for 30 min with natural products at various final concentrations in 1 μl DMSO; and subsequently the reaction was initiated by adding Bz-nKRR-AMC to 250 μM. For *K*i determination, the assay was performed with different final concentrations of the inhibitors and substrate. Briefly, the Zika protease at 50 nM was preincubated with the inhibitor at different concentrations for 30 min at 37°C. Subsequently, the reaction was initiated by addition of the corresponding concentration series of the substrate. All measurements were performed in triplicate and data are presented as mean ± SD. The *K*i was obtained by fitting in the non-competitive inhibition mode with GraphPad Prism 7.0, with an equation: Vmaxinh = Vmax/(1+I/Ki), while I is the concentration of inhibitor [61].

### Molecular docking

To gain insight into structural details of the binding pocket, we docked all six active natural products to the crystal structure (PDB code of 5LC0) of Zika NS2B-NS3pro in complex with an active site inhibitor cn-716 [34]. The chemical structures of the compounds were downloaded from ZINC (http://zinc.docking.org) and ChemicalBook database (http://www.chemicalbook.com) respectively. Subsequently the structures were geometrically optimized with Avogadro [62]. The partial charges of all atoms in small compounds and Zika NS2B-NS3pro were assigned with Gasteiger-Marsili charges, and non-polar hydrogen atoms were merged into the appropriate heavy atoms with AutoDockTools [47]. AutoDock software (Version 4.2) was utilized to dock six compounds to the crystal structure of Zika NS2B-NS3pro. The grid box was set with 74 × 70 × 66 (x,y,z axis) with the default 0.375Å spacing. The initial population size was set to 300, and the number of energy evaluations was set to 25,000,000, and number of docking runs was set as 150. The results were clustered with each cluster having a tolerance of 2 Å. The complexes with the lowest energy were selected for analysis and display.

## Supporting information

S1 Fig Construction, expression and purification of Zika NS2B-NS3pro complexes. (A) Sequence alignment between NS2B (48–100) of the Dengue and Zika viruses with the transmembrane region removed. The red arrow is used to indicate the region with significant sequence variations. (B) Sequence alignment between NS3pro (14–185) of Dengue and Zika viruses. (C) SDS PAGE of the samples at different purification steps of linked Zika NS2B-NS3pro: column 1: molecular weight makers; column 2: linked Zika NS2B-NS3pro; column 3: linked Zika NS2B-NS3pro with the His-tag removed by the thrombin beads followed by binding to an excess amount of Ni^2+^-beads. (D) SDS PAGE of the samples at different purification steps of unlinked Zika NS2B-NS3pro: column 1: molecular weight makers; column 2: unlinked Zika NS2B-NS3pro; column 3: unlinked Zika NS2B-NS3pro with the His-tag removed by the thrombin beads followed by binding to an excess amount of Ni^2+^-beads. (E) SDS PAGE of the samples at different purification steps of unlinked Zika NS2B (48–74)-NS3pro: column 1: molecular weight makers; column 2: unlinked Zika NS2B (48–74)-NS3pro. Due to the small sizes of NS2B(48–100) and NS2B(48–74), they diffused and thus could not be seen in SDS PAGE. (F) The exact same sample for SDS PAGE shown in (D) was analysed by high pressure liquid chromatography (HPLC) on a reverse-phase (RP) C4 column, which clearly showed the presence of two peaks: one eluted out at 8.1 min for NS2B and another at 27.4 min for NS3pro.
(TIF)

S2 Fig NMR characterization of selectively labeled NS3pro and NS2B of Zika NS2B-NS3pro. (A) ^1^H-^15^N HSQC spectrum of ^15^N-labeled Zika NS3pro in complex with unlabeled Zika NS2B at a protein concentration of 30 μM. Pink arrows are used to indicate the HSQC peaks of Trp50, Trp69, Trp83 and Trp89 side chains in NS3pro. (B) ^1^H-^15^N HSQC spectrum of ^15^N-labeled Zika NS2B in complex with unlabeled Zika NS3pro at a protein concentration of 30 μM, in which only HSQC peaks of non-Pro residues of NS2B are detectable. Pink arrow is used to indicate the HSQC peak of Trp61 side chain in NS2B. (C) Simulated ^1^H-^15^N HSQC spectrum of Dengue-2 NS2B in complex with Dengue NS3pro, which was generated by extracting chemical shifts of amide nitrogen-15 and proton atoms of Dengue-2 NS2B deposited in BMRB (Entry ID of 19080).
(TIF)

S3 Fig Sequence alignment of NS2B (48–100) of Zika and four serotype Dengue viruses. The red arrow is used to indicate the region with significant sequence variations between Zika and Dengue.
(TIF)

S4 Fig Catalytic properties of Zika NS2B-NS3pro. (A) The tracings of fluorescence intensity within 3 min for three different substrates cleaved by the linked Zika NS2B-NS3pro complex: Bz-nKRR-AMC, Boc-GRR-AMC and Boc-GKR-AMC; as well as three assay buffers without the protease. Fluorescence intensity is reported in arbitrary units. (B) Enzymatic activities of linked (blue) and unlinked Zika NS2B-NS3pro complexes at different pH values. (C) Enzymatic activities of linked (blue) and unlinked (red) Zika NS2B-NS3pro complexes in 50 mM Tris buffer at pH 8.5 with additional addition of NaCl at 0, 20, 40, 60, 80, 100, 125, 150, 200, 250 mM. (D) Enzymatic activities of linked (blue) and unlinked (red) Zika NS2B-NS3pro complexes in 50 mM Tris buffer at pH 8.5 with additional presence of glycerol at 0, 5%, 10%, 15%, 20%, 25%, 30%, 35%, 40%. (E) Lineweaver-Burke plots for determine Km values of the unlinked Zika NS2B-NS3pro in different assay buffers. [S] is the substrate concentration; v is the initial reaction rate.
(TIF)

S5 Fig Inhibition of Zika NS2B-NS3pro by six natural products. (A) Inhibitory data used for fitting IC_50_ values for Myricetin, Quercetin, Luteolin and Curcumin. (B) Inhibitory data used for fitting IC_50_ values for Isorhamnetin and Apigenin.
(TIF)

S6 Fig Different properties of the binding pockets of the Zika and Dengue NS2B-NS3pro complexes for natural products. (A) The electrostatic potential surface of the docking model for the Zika NS2B-NS3pro (PDB code of 5LC0) in complex with Myricetin (yellow) and Curcumin (cyan), inclusive of its active site inhibitor cn-716 in spheres. (B) Expanded allosteric pocket bound with Myricetin (yellow) and Curcumin (cyan). (C) The electrostatic potential surface of the crystal structure (PDB code of 3U1I) of Dengue-2 NS2B-NS3pro determined with an active site inhibitor Bz-nKRR (in spheres), which was previously used to build docking models with flavonoids including Myricetin and Quercetin. (D) Expanded allosteric pocket of Dengue-2 NS2B-NS3pro. The yellow ellipsoid is used to indicate the pocket previously identified for binding flavonoids including Myricetin and Quercetin.
(TIF)
