# Supplementary figures and images for: Solution conformations of Zika NS2B-NS3pro and its inhibition by natural products from edible plants
Source: PLoS One. 2017 Jul 10;12(7):e0180632. doi: 10.1371/journal.pone.0180632 (PMC5503262; doi:10.1371/journal.pone.0180632)

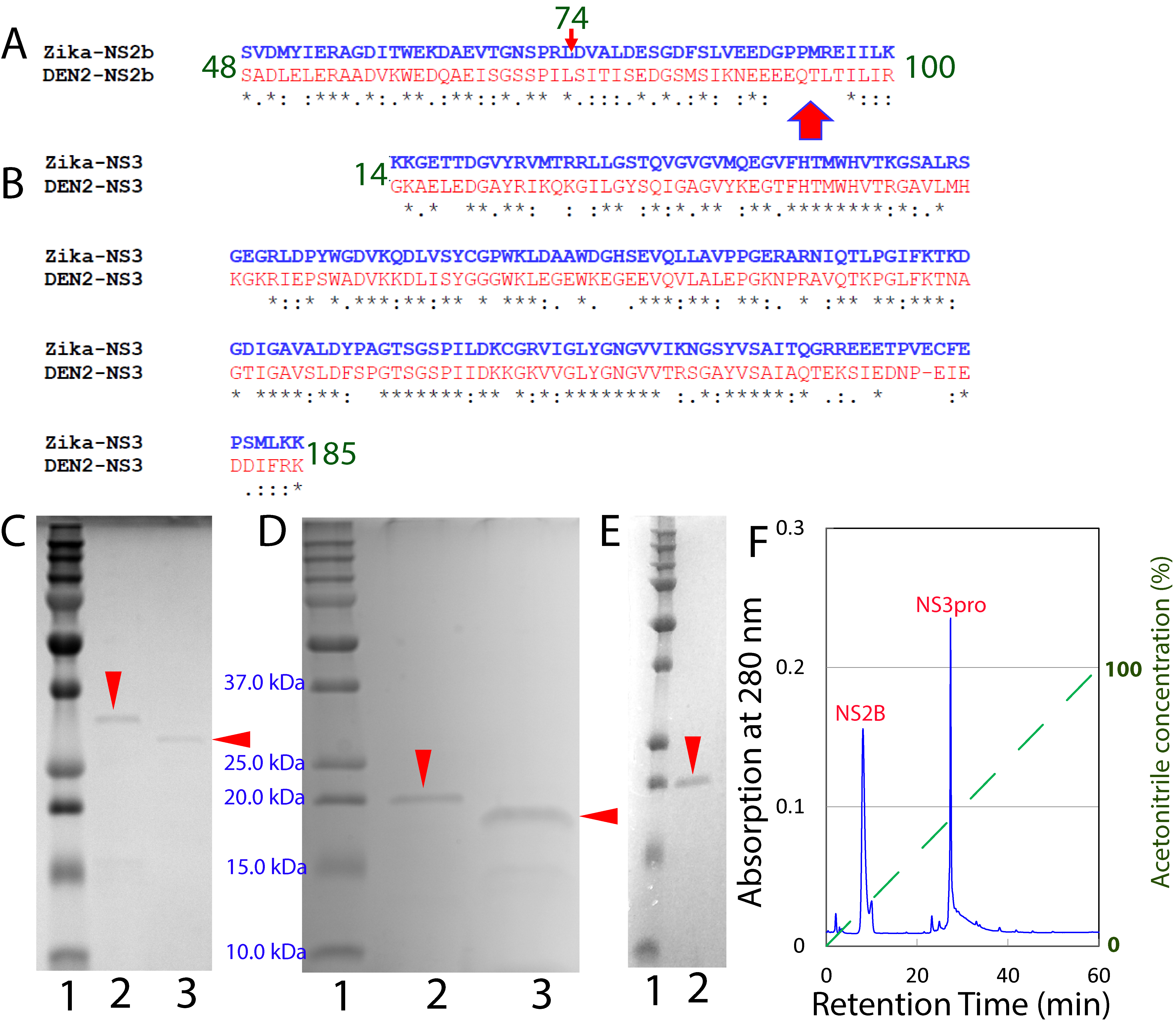

Supplement: S1 Fig — (A) Sequence alignment between NS2B (48–100) of the Dengue and Zika viruses with the transmembrane region removed. The red arrow is used to indicate the region with significant sequence variations. (B) Sequence alignment between NS3pro (14–185) of Dengue and Zika viruses. (C) SDS PAGE of the samples at different purification steps of linked Zika NS2B-NS3pro: column 1: molecular weight makers; column 2: linked Zika NS2B-NS3pro; column 3: linked Zika NS2B-NS3pro with the His-tag removed by the thrombin beads followed by binding to an excess amount of Ni2+-beads. (D) SDS PAGE of the samples at different purification steps of unlinked Zika NS2B-NS3pro: column 1: molecular weight makers; column 2: unlinked Zika NS2B-NS3pro; column 3: unlinked Zika NS2B-NS3pro with the His-tag removed by the thrombin beads followed by binding to an excess amount of Ni2+-beads. (E) SDS PAGE of the samples at different purification steps of unlinked Zika NS2B (48–74)-NS3pro: column 1: molecular weight makers; column 2: unlinked Zika NS2B (48–74)-NS3pro. Due to the small sizes of NS2B(48–100) and NS2B(48–74), they diffused and thus could not be seen in SDS PAGE. (F) The exact same sample for SDS PAGE shown in (D) was analysed by high pressure liquid chromatography (HPLC) on a reverse-phase (RP) C4 column, which clearly showed the presence of two peaks: one eluted out at 8.1 min for NS2B and another at 27.4 min for NS3pro. (TIF) [file pone.0180632.s001.tif]

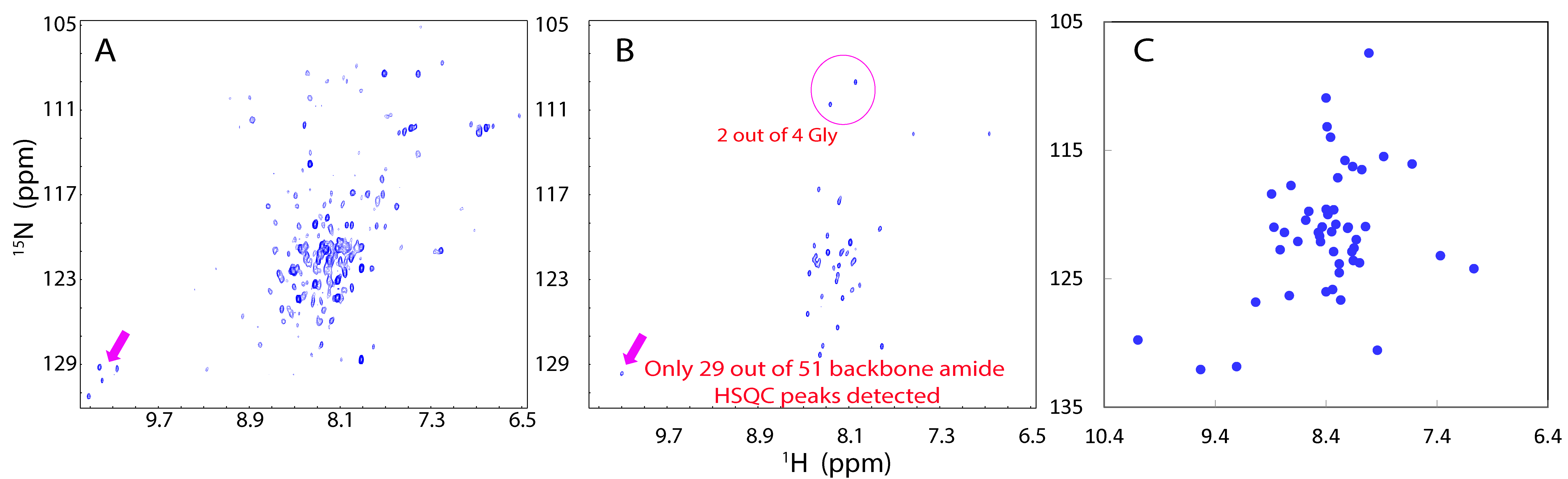

Supplement: S2 Fig — (A) 1H-15N HSQC spectrum of 15N-labeled Zika NS3pro in complex with unlabeled Zika NS2B at a protein concentration of 30 μM. Pink arrows are used to indicate the HSQC peaks of Trp50, Trp69, Trp83 and Trp89 side chains in NS3pro. (B) 1H-15N HSQC spectrum of 15N-labeled Zika NS2B in complex with unlabeled Zika NS3pro at a protein concentration of 30 μM, in which only HSQC peaks of non-Pro residues of NS2B are detectable. Pink arrow is used to indicate the HSQC peak of Trp61 side chain in NS2B. (C) Simulated 1H-15N HSQC spectrum of Dengue-2 NS2B in complex with Dengue NS3pro, which was generated by extracting chemical shifts of amide nitrogen-15 and proton atoms of Dengue-2 NS2B deposited in BMRB (Entry ID of 19080). (TIF) [file pone.0180632.s002.tif]

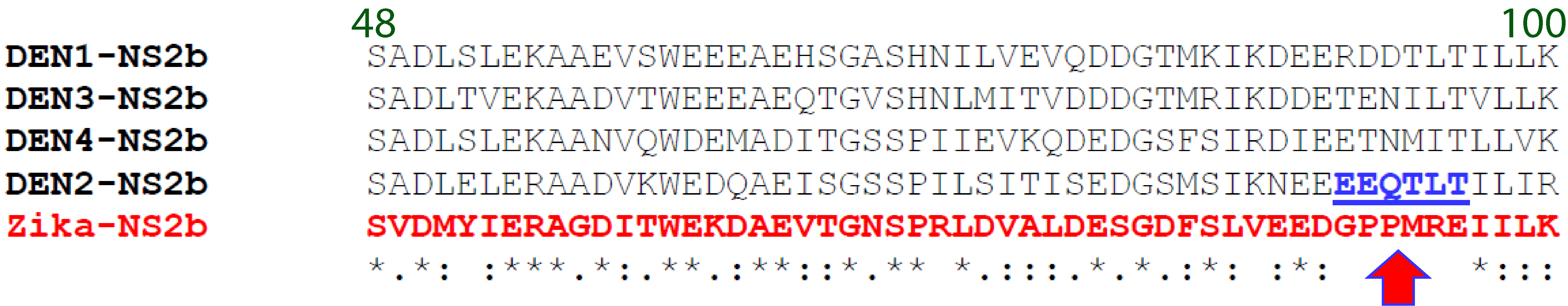

Supplement: S3 Fig — The red arrow is used to indicate the region with significant sequence variations between Zika and Dengue. (TIF) [file pone.0180632.s003.tif]

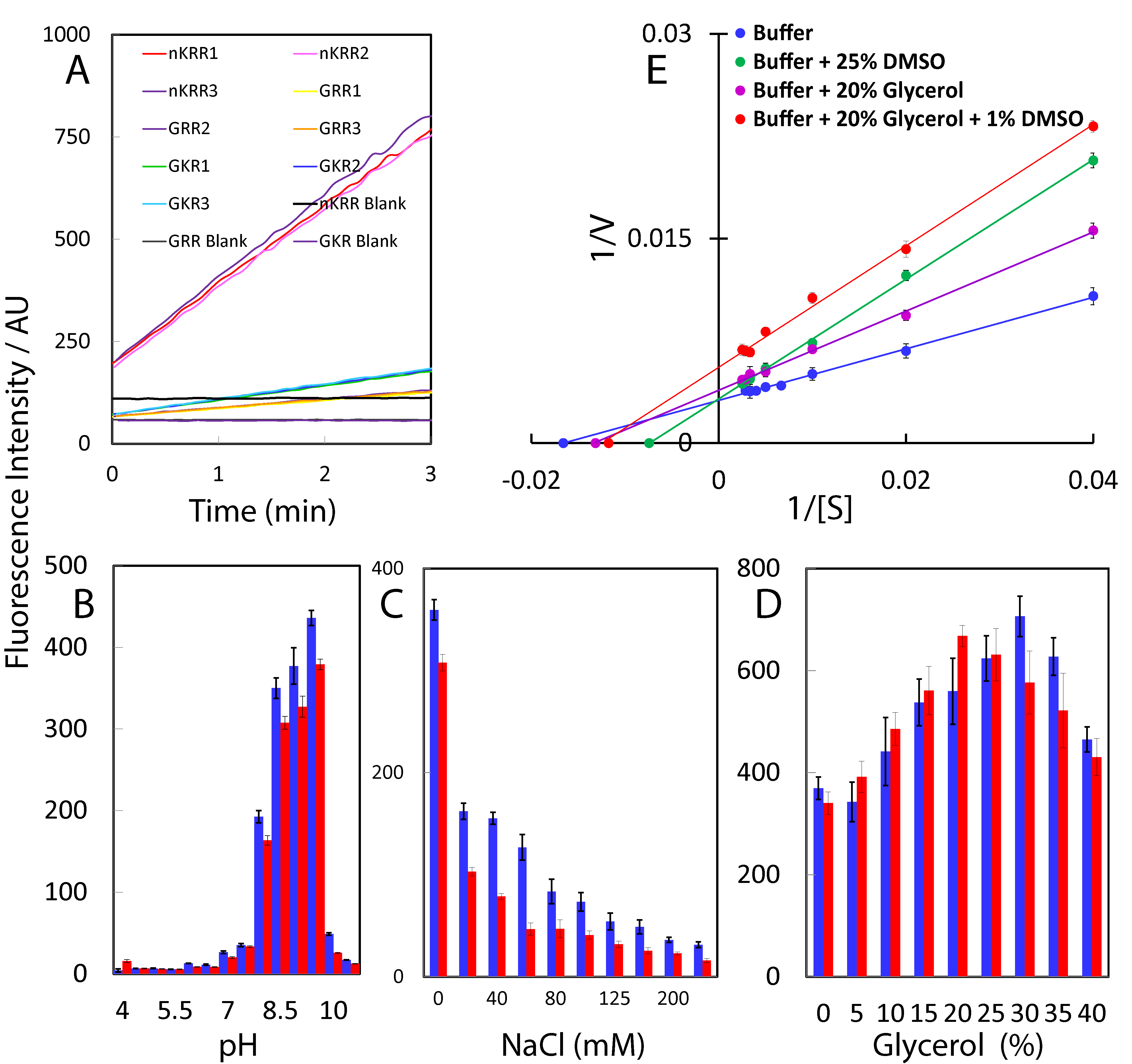

Supplement: S4 Fig — (A) The tracings of fluorescence intensity within 3 min for three different substrates cleaved by the linked Zika NS2B-NS3pro complex: Bz-nKRR-AMC, Boc-GRR-AMC and Boc-GKR-AMC; as well as three assay buffers without the protease. Fluorescence intensity is reported in arbitrary units. (B) Enzymatic activities of linked (blue) and unlinked Zika NS2B-NS3pro complexes at different pH values. (C) Enzymatic activities of linked (blue) and unlinked (red) Zika NS2B-NS3pro complexes in 50 mM Tris buffer at pH 8.5 with additional addition of NaCl at 0, 20, 40, 60, 80, 100, 125, 150, 200, 250 mM. (D) Enzymatic activities of linked (blue) and unlinked (red) Zika NS2B-NS3pro complexes in 50 mM Tris buffer at pH 8.5 with additional presence of glycerol at 0, 5%, 10%, 15%, 20%, 25%, 30%, 35%, 40%. (E) Lineweaver-Burke plots for determine Km values of the unlinked Zika NS2B-NS3pro in different assay buffers. [S] is the substrate concentration; v is the initial reaction rate. (TIF) [file pone.0180632.s004.tif]

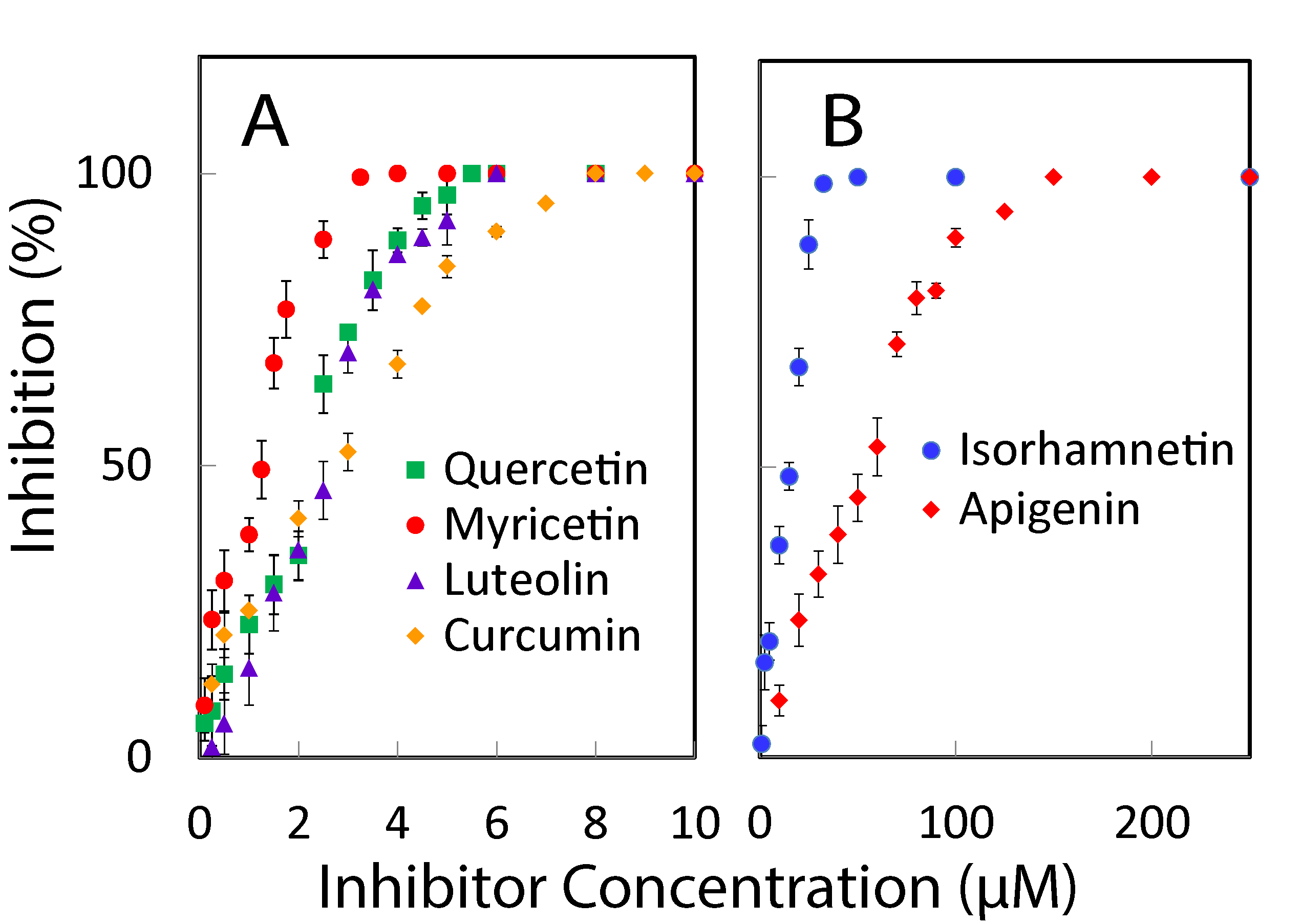

Supplement: S5 Fig — (A) Inhibitory data used for fitting IC50 values for Myricetin, Quercetin, Luteolin and Curcumin. (B) Inhibitory data used for fitting IC50 values for Isorhamnetin and Apigenin. (TIF) [file pone.0180632.s005.tif]

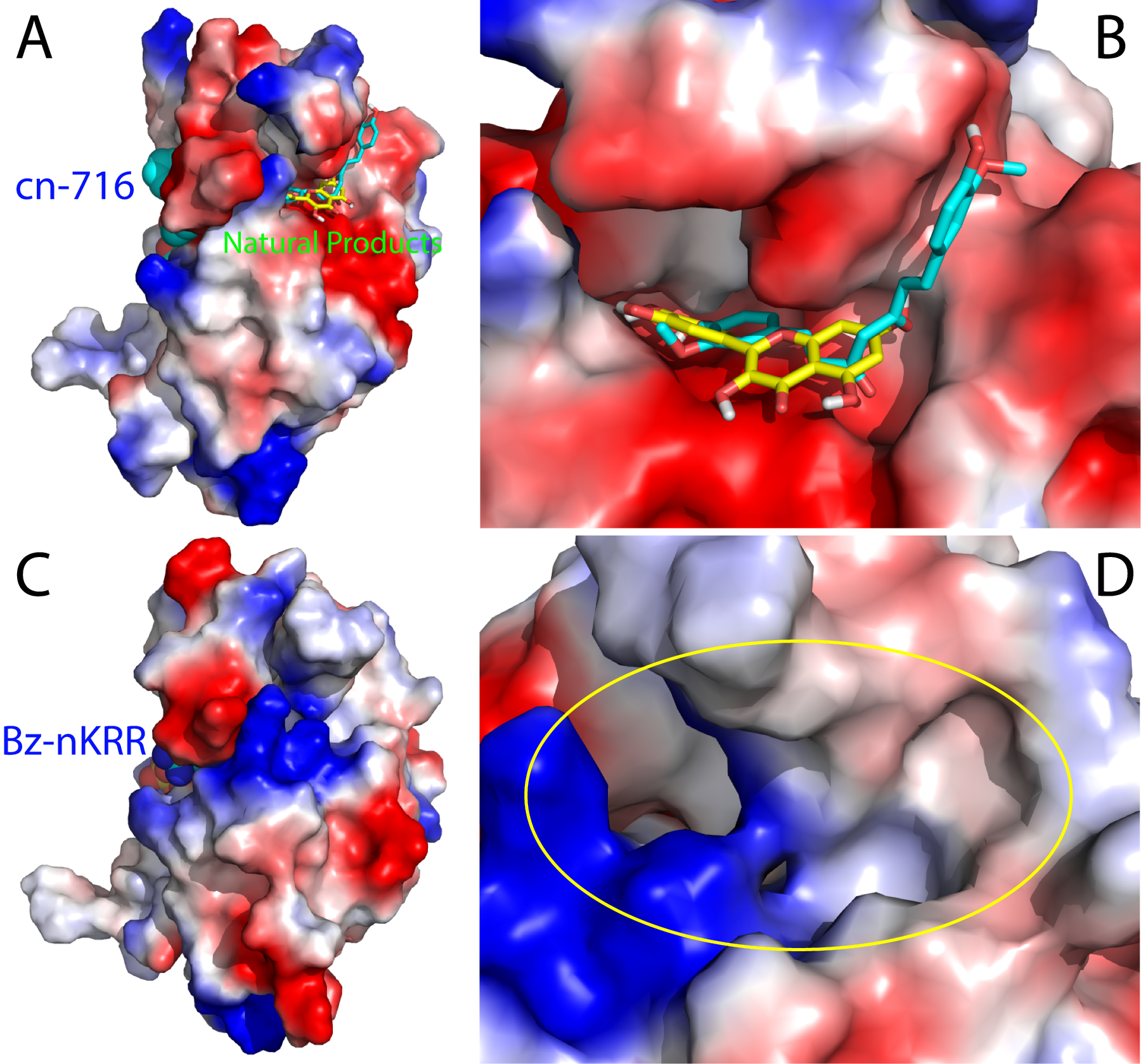

Supplement: S6 Fig — (A) The electrostatic potential surface of the docking model for the Zika NS2B-NS3pro (PDB code of 5LC0) in complex with Myricetin (yellow) and Curcumin (cyan), inclusive of its active site inhibitor cn-716 in spheres. (B) Expanded allosteric pocket bound with Myricetin (yellow) and Curcumin (cyan). (C) The electrostatic potential surface of the crystal structure (PDB code of 3U1I) of Dengue-2 NS2B-NS3pro determined with an active site inhibitor Bz-nKRR (in spheres), which was previously used to build docking models with flavonoids including Myricetin and Quercetin. (D) Expanded allosteric pocket of Dengue-2 NS2B-NS3pro. The yellow ellipsoid is used to indicate the pocket previously identified for binding flavonoids including Myricetin and Quercetin. (TIF) [file pone.0180632.s006.tif]
